# Asymptomatic Endemic *Chlamydia pecorum* Infections Reduce Growth Rates in Calves by up to 48 Percent

**DOI:** 10.1371/journal.pone.0044961

**Published:** 2012-09-14

**Authors:** Anil Poudel, Theodore H. Elsasser, Kh. Shamsur Rahman, Erfan U. Chowdhury, Bernhard Kaltenboeck

**Affiliations:** 1 Department of Pathobiology, College of Veterinary Medicine, Auburn University, Auburn, Alabama, United States of America; 2 Bovine Functional Genomics Laboratory, United States Department of Agriculture - Agricultural Research Service, Beltsville, Maryland, United States of America; University of California Merced, United States of America

## Abstract

Intracellular *Chlamydia* (*C.*) bacteria cause in cattle some acute but rare diseases such as abortion, sporadic bovine encephalomyelitis, kerato-conjunctivitis, pneumonia, enteritis and polyarthritis. More frequent, essentially ubiquitous worldwide, are low-level, asymptomatic chlamydial infections in cattle. We investigated the impact of these naturally acquired infections in a cohort of 51 female Holstein and Jersey calves from birth to 15 weeks of age. In biweekly sampling, we measured blood/plasma markers of health and infection and analyzed their association with clinical appearance and growth in dependence of chlamydial infection intensity as determined by mucosal chlamydial burden or contemporaneous anti-chlamydial plasma IgM. *Chlamydia* 23S rRNA gene PCR and *ompA* genotyping identified only *C. pecorum* (strains 1710S, Maeda, and novel strain Smith3v8) in conjunctival and vaginal swabs. All calves acquired the infection but remained clinically asymptomatic. High chlamydial infection associated with reduction of body weight gains by up to 48% and increased conjunctival reddening (*P*<10^−4^). Simultaneously decreased plasma albumin and increased globulin (*P*<10^−4^) suggested liver injury by inflammatory mediators as mechanisms for the growth inhibition. This was confirmed by the reduction of plasma insulin like growth factor-1 at high chlamydial infection intensity (*P*<10^−4^). High anti-*C. pecorum* IgM associated eight weeks later with 66% increased growth (*P* = 0.027), indicating a potential for immune protection from *C. pecorum*-mediated growth depression. The worldwide prevalence of chlamydiae in livestock and their high susceptibility to common feed-additive antibiotics suggests the possibility that suppression of chlamydial infections may be a major contributor to the growth promoting effect of feed-additive antibiotics.

## Introduction

Obligate intracellular bacteria of the phylum *Chlamydiae* infect virtually every eukaryotic organism, from single-celled amoebae to multicellular hosts including vertebrates [1]. The family *Chlamydiaceae* comprises the single genus *Chlamydia* (*C.*) which encompasses nine species that cause the majority of chlamydial diseases in mammals and birds [2]. In cattle, the two species, *C. abortus* and *C. pecorum*, are routinely detected in acute infections with distinct clinical symptoms such as fertility disorders and abortion, mastitis, sporadic encephalomyelitis, kerato-conjunctivitis, pneumonia, enteritis and polyarthritis [3]–[11]. However, besides these infrequent acute infections many more asymptomatic chlamydial infections can be detected in livestock [12], particularly after introduction of PCR diagnostics and commercially available ELISA assays [13], [14]. It has been understood for a long time [15] that a large percentage of cattle cohorts are *Chlamydia*-positive when just a few animals of these cohorts are randomly sampled [16], [17], [18], albeit at different prevalence rates in individual animals ranging from quite low (<5%) to high (50–100%) [12]. Even higher herd prevalence typically associates with frequent sampling of many animals and high assay quality [14], [19]–[23], large cohort size and population density [24], open and low-quality herd management, poor hygiene, natural siring [25], and nutritional deficiencies resulting in metabolic disorders [26]. Thus, high prevalence in essence associates with all factors that favor susceptibility to, and transmission of, chlamydial infections, and with effective methods to detect them.

The few cases of severe or fatal chlamydial disease, in particular circumstances that favor transmission such as herd abortions, have always been considered the “tip of the iceberg” [27], but these rare diseases are of little economic consequence. However, the health impact and economic consequences of the ubiquitous asymptomatic chlamydial infections in cattle have largely remained unknown. Only few studies have addressed this question, but they all significantly associate clinically asymptomatic chlamydial infections with decreased herd health and performance. Wehrend et al. [26] found a high risk for ovarian cysts and reproductive disorders if chlamydial antigen was present in the uterus of dairy cows, and Jaeger et al. [28] and Reinhold et al. [29] showed that latent chlamydial respiratory infection associated with airway obstruction and pulmonary inflammation in calves aged 2–7 months. Kemmerling et al. [25] found lower milk yield and reproductive performance in dairy herds that tested positive for chlamydial infection. While these studies demonstrated an association between asymptomatic chlamydial infection and health, they could not elucidate the cause-effect relationship, i.e. if chlamydial infection caused the health disorder or if the health disorder made the animals more susceptible to chlamydial infection. However, in an interventional field study using vaccination against *C. abortus* and *C. pecorum*, Biesenkamp et al. [30] unequivocally demonstrated causality of latent chlamydial infection when *Chlamydia*-vaccinated animals showed a highly significant decline in milk somatic cell counts over mock-vaccinated animals. In an experimental study, DeGraves at al. [3] showed a causal effect of chlamydial infection on reduction of fertility in heifers after *C. abortus* challenge without any clinical disease symptoms.

Similar to adult cattle, little is known about the health effects of widespread clinically asymptomatic chlamydial infections in calves. Calves are typically born free of chlamydiae [24], but may also be born infected after in utero infection [31], and become again infected within the first weeks of life. Jee at al. [24] reported a 61% prevalence of chlamydial infection in apparently healthy young calves.

The objective of the present investigation was to quantify the impact of these infections in a comprehensive prospective study. We followed a cohort of female calves from birth to 15 weeks of age, and found that they all became asymptomatically infected with *C. pecorum*. Thus, rather than associating presence or absence of chlamydial infection with changed health, we analyzed if changed intensity of chlamydial infection resulted in different health outcomes. In addition to parameters of chlamydial infection and clinical appearance, whole blood and plasma markers for health and growth were evaluated. In the absence of overt disease, we focused on physiological parameters that would be negatively affected by responses to chronic inflammation, in particular body weight and its change over time, the economically most important health parameter in juvenile cattle. We show here that subclinical infection by *Chlamydia pecorum* reduces growth rates and body weight, and find strong support that anti-*C. pecorum* immunity after peak infection protects calves from subsequent losses in body weight.

## Results

### Development of calves

**Figure 1:**
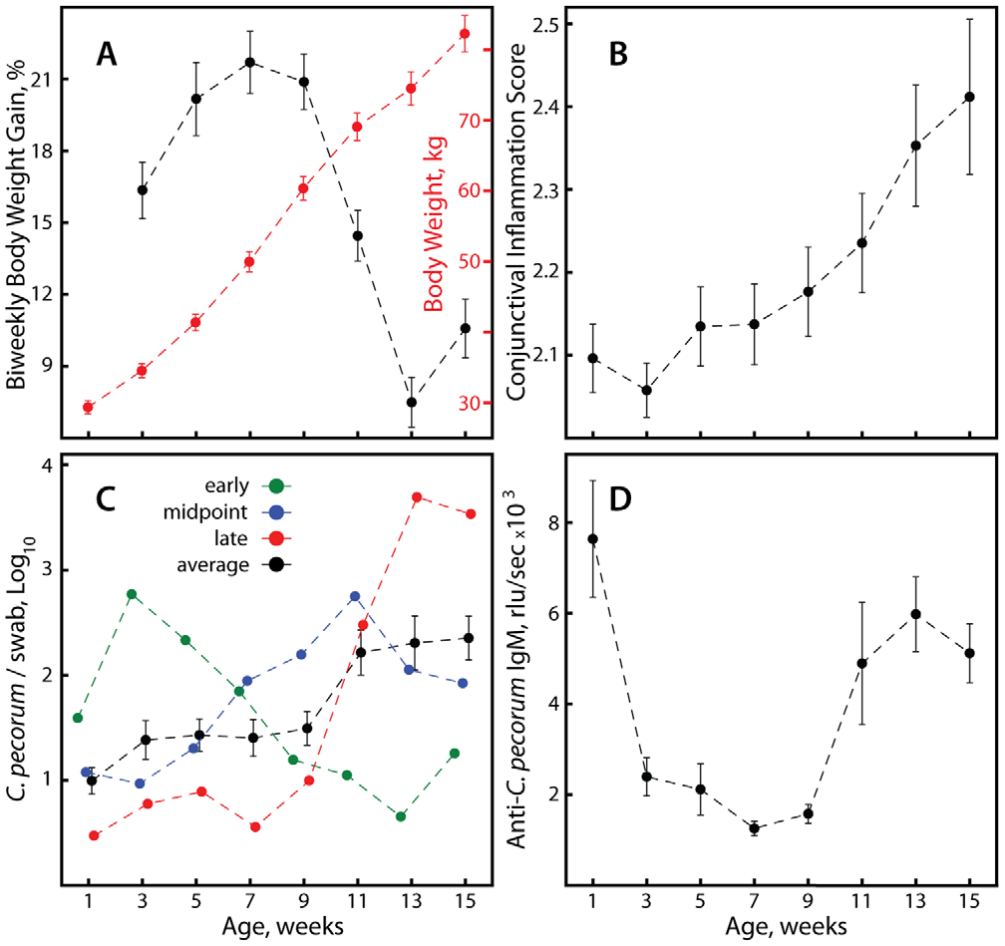
Development of calves and chlamydial infection. The progression over the sampling period is shown (n = 51). (A) Body weight gain over successive 2-week periods and absolute body weight. (B) Conjunctival inflammation as expressed by an arbitrary score from 1–4 for redness, with 2 for normal pink coloration of the conjunctiva. (C) Average *C. pecorum* genomes per cytobrush swab detected by *Chlamydia* spp. 23S rRNA gene real-time FRET PCR. Early, midpoint, or late indicates peak *C. pecorum* infection before week 9, in week 9 or 11, or in week 13 or 15. For clarity, only the mean of all calves is shown with error bars. (D) Anti-*C. pecorum* IgM antibodies as determined by chemiluminescent ELISA using a lysate antigen of *C. pecorum* elementary bodies. Data are shown as means ± SEM.

Over a 6 month period, a total of 26 Jersey and 25 Holstein female calves were enrolled in the study immediately after birth. If possible, plasma and swab samples were collected prior to the first colostrum feeding and calves were further sampled in two-week intervals from one week to fifteen weeks of age. The average body weight in the first week of life was 29.97±0.94 (SEM) kg (Jersey 26.14±0.79 kg, Holstein 33.81±1.34 kg; *P*<10^−4^), and at 15 weeks 82.86±2.57 kg (Jersey 70.66±2.37 kg, Holstein 95.55±2.99 kg; *P*<10^−4^) (Fig. 1A). The average growth rate expressed as percent body weight difference relative to the body weight two weeks earlier was 15.22±0.48%, without significant difference between the breeds. As evident in Fig. 1A, biweekly body weight gain declined from the maximum of 21.7% in week 7 to a minimum of 7.5% in week 13, presumably due in part to abrupt weaning at 8 weeks of age and the switch to roughage feeding with low nutrient density [32].

The clinical appearance of all calves during the entire study period was normal, without obvious signs of clinical disease. Similarly, complete and differential blood cell counts as well as plasma albumin, globulin, iron, and IGF-1 did not exceed normal ranges. However, as shown in Fig. 1B, the arbitrary score for redness and inflammation of the conjunctiva (1 = anemic, 2 = normal pink, 3 = pronounced red, 4 = deep red) continuously and highly significantly increased from less than 2.1 in weeks 1 and 3 to 2.4 in week 15 (*P*<0.005).

### Chlamydial infection

Out of 43 precolostral conjunctival and vaginal cytobrush swab samples obtained immediately after birth from the total of 51 calves in the study, 27 were negative for chlamydial DNA in the *Chlamydia* spp. 23S rRNA gene real-time PCR, but 16 calves were positive at low copy number below 10 chlamydial genomes in either conjunctival or vaginal swab, or both (n = 8, 5, and 3, respectively). Some calves (n = 16, 37%) at birth were free of PCR or serological evidence (anti-*C. pecorum* IgM) of chlamydial infection, while all others either showed only PCR (n = 8, 19%) or serological evidence (n = 11, 28%) of chlamydial exposure, or both (n = 8, 19%). Eventually, all calves in the study became *Chlamydia* spp. PCR-positive as well as developed anti-*C. pecorum* IgM. The data from pre-colostrum sampling were used to establish pre- or postnatal chlamydial infection, but were not used in subsequent repetitive analyses of the calves.

From all 51 calves included in the study, a total of 816 conjunctival and vaginal specimens were collected in 2-week intervals between 1 to 15 weeks of age, and 606 (74.3%) of these specimens were positive in the *Chlamydia* spp. 23S rRNA gene PCR. The average chlamydial load per positive conjunctival swab was 43 genomes (range 1–65,800; antilog), and of vaginal swabs 227 (range 1–1,771,600; antilog). The only chlamydial species detected was *C. pecorum* in the *Chlamydia* spp. 23S rRNA gene PCR.

Of the 408 paired conjunctival-vaginal calf specimens, 64 were negative in both swabs, 50 were positive only in the conjunctival swab, 32 only in the vaginal swab, and 262 positive in both, with no breed difference. Based on the statistically identical positivity of both sampling sites (Chi square test), but the lower conjunctival load (*P*<10^−4^), we calculated a mean-adjusted overall chlamydial burden per calf and sampling time as described in methods, referred thereafter as *C. pecorum*/swab. Typically, both conjunctival and vaginal specimens became very low positive at one sampling time point, remained low for two to four weeks, then increased to peak positivity and declined again to negativity over two to four weeks. Based on the infection kinetics, we categorized the calves into three approximately equal groups as early (1–5 weeks: n = 13), midpoint (7–11 weeks: n = 20) or late (13–15 weeks: n = 18) peak *C. pecorum*-infected (Fig. 1C). Average infection intensity was independent of breed and increased significantly with the age of the calves (e.g., week 7 vs. week 15 = 24 vs. 225 genomes/swab; *P*<0.001) particularly after weaning at 8 weeks when they were transferred from individual hutches to a common pasture. Some of the calves with an early peak infection before week 5 had a recurrence or increase of the infection towards the end of sampling in week 15.

*C. pecorum ompA* real-time PCR of 31 selected specimens from 19 calves distributed along the complete sampling period confirmed *C. pecorum* [33]–[37]. However, sequencing of the amplification products identified three distinct strains with the *ompA* genotypes 1710S (GenBank Accession # M73033.1), Maeda (GenBank Accession # AB512085.1), and the novel *ompA* genotype Smith3v8 (GenBank Accession # JX272924). Over the first eight months of specimen collection, only genotype Smith3v8was identified in *C. pecorum*-positive swabs. This strain disappeared within a two-week interval and was replaced by strains 1710S and Maeda in the last 3 sampling months. In the strain transition period, mixed infections with combinations of Smith3v8 and Maeda and/or 1710S were identified in four calves by dual peaks at polymorphic positions in the *ompA* amplicon sequences. Three calves also were infected with different strains in conjunctiva and vagina, and three calves over time showed repeated infection peaks with different strains before and after the 2-week strain transition period, while such repeated peaks prior to transition were only caused by strain Smith3v8.

### Anti-*C. pecorum* IgM

From 40 calves sampled that had presumably received no colostrum, a complete absence of plasma IgM antibodies against *C. pecorum* indicated that only 21 truly had been sampled immediately after birth before first suckling, while 19 showed variable levels of anti-*C. pecorum* IgM. Mean plasma anti-*C. pecorum* IgM were highest with 7569 rlu/sec at 1 week of age, then dropped precipitously to a minimum of 1,256 rlu/sec early sampling time points (Fig. 1D, *P*<10^−4^), suggesting a rapid disappearance of passively acquired IgM antibodies [38], [39], [40]. After week 7, IgM antibody levels rose again, presumably due to an emerging antibody response to chlamydial infection, with a secondary peak of 5,978 rlu/sec anti-*C. pecorum* IgM in week 13. Similar to chlamydial infection, the kinetics of antichlamydial antibody levels also varied between calves. While in some calves, antibody levels remained at or dropped to zero for several weeks after week 1 (n = 15), others showed only a minor decline followed by steady or increasing antibody levels (n = 36). Based on the week-7 antibody minimum, to lessen the potential influence of passive immunity, we performed subsequent analyses with data only from week 7 to 15, and utilized the week 1-to-week 5 dataset for confirmation of results.

### Physiological effects of *C. pecorum* infection

To determine if *C. pecorum* infection influenced health and weight gain of the calves, we categorized the data of all calves from week 7 through week 15 into 2 or 3 ordinal groups based on parameters of contemporaneous chlamydial exposure, i.e. *C. pecorum* load (*C. pecorum*/swab) or anti-*C. pecorum* IgM, the primary and early response antibody isotype [24]. These 255 observations were separated into 2 or 3 equal groups of low or high, or low, intermediate, or high parameter values. Analysis of these groups revealed significant differences in biweekly weight gains, conjunctival inflammation, and several plasma markers that correlated with *C. pecorum* load or anti-*C. pecorum* IgM (Table 1, 2). Most prominently, high chlamydial loads or high antichlamydial antibodies highly significantly associated with reduced weight gain and increased conjunctival inflammation (Table 1). Animals with high chlamydial load showed 13.77% biweekly weight gain as compared to animals with low load with a 16.26% weight gain, a 15% reduction in the growth of calves with high chlamydial infection (*P* = 0.047). This association was even more pronounced in 3-category analysis of chlamydial load groups (*P* = 0.003), or when animals were categorized by antibody levels. At the highest difference at 3 antibody categories, animals with low anti-*C. pecorum* plasma antibodies showed an 18.90% biweekly weight gain, while calves with high antibody levels gained only 10.91% body weight over 2 weeks, a 42% growth rate reduction (*P*<10^−4^). Conjunctival inflammation scores followed a similarly significant, but reverse pattern in which high chlamydial load or anti-chlamydial antibodies associated with high inflammation. This suggests a strong association between inflammatory response to chlamydial infection and the rate of weight gain in which high inflammation correlates with low weight gains in these calves.

**Table 1:** Physiological and chlamydial infection markers categorized by *C. pecorum* load and plasma anti-*C. pecorum* IgM.

Grouping Parameter	Category#	N	Biweekly Body Weight Gain, %		Conjunctival Inflammation Score		*C. pecorum*/swab, Log_10_		Anti-*C. pecorum* IgM, rlu/sec	
			Mean	95% CI	Mean	95% CI	Mean	95% CI	Mean	95% CI
***C. pecorum*** ** load**	**Low**	128	16.26^a^	14.57–17.95	2.15^A^	2.09–2.21	0.69^A^	0.59–0.80	2,151^A^	1,765–2,538
	**High**	127	13.77	11.98–15.56	2.39	2.28–2.49	3.24	3.07–3.41	5,408	4,073–6,742
	**Low**	85	16.46^A^	14.51–18.41	2.14^A^	2.07–2.22	0.34^A^^,^ B	0.26–0.42	2,034^A^	1,619–2,450
	**Intermediate**	85	16.35^A^	13.98–18.71	2.21^A^	2.12–2.31	1.79^A^	1.69–1.89	2,657^A^	1,974–3,339
	**High**	85	12.25	10.23–14.27	2.45	2.32–2.58	3.75	3.57–3.92	6,629	4,751–8,506
**Anti-** ***C. pecorum*** ** IgM**	**Low**	128	17.53^A^	15.78–19.28	2.14^A^	2.08–2.20	1.46^A^	1.26–1.66	878^A^	753–1,003
	**High**	127	12.49	10.85–14.13	2.39	2.29–2.50	2.45	2.16–2.75	6,691	5,443–7,938
	**Low**	85	18.90^A^^,^ b	16.68–21.13	2.11^A^	2.04–2.17	1.52^A^	1.26–1.78	463^A^^,^ B	362–563
	**Intermediate**	85	15.24^A^	13.40–17.08	2.20^A^	2.11–2.29	1.47^A^	1.21–1.73	2,186^A^	2,055–2,317
	**High**	85	10.91	8.87–12.96	2.49	2.36–2.63	2.88	2.52–3.24	8,670	6,950–10,390

# *C. pecorum* load: low <1.79, high ≥1.79; low <1.02, intermediate <2.62, high ≥2.62; anti-*C. pecorum* IgM: low <2173, high ≥2173; low <1270, intermediate <3636, high ≥3636.

Significant differences are indicated by.

a from “High” at *P*<0.05;

A from “High” at *P*<0.01;

b from “Intermediate” at *P*<0.05;

B from “Intermediate” at *P*<0.01.

**Table 2:** Plasma markers categorized by *C. pecorum* load and anti-*C. pecorum* plasma IgM category.

Grouping Parameter	Category#	N	Albumin, g/dL		Globulin, g/dL		IGF-1, ng/mL	
			Mean	95% CI	Mean	95% CI	Mean	95% CI
***C. pecorum*** ** load**	**Low**	128	3.31^A^	3.27–3.34	3.00^A^	2.91–3.09	105.32^a^	93.07–117.57
	**High**	127	3.17	3.13–3.22	3.32	3.22–3.42	87.57	77.11–98.03
	**Low**	85	3.30^A^	3.26–3.35	2.98^A^	2.88–3.09	107.46^A^	92.22–122.70
	**Intermediate**	85	3.29^A^	3.24–3.34	3.08^A^	2.98–3.19	101.36^a^	87.24–115.48
	**High**	85	3.13	3.07–3.19	3.41	3.29–3.54	80.63	68.24–93.01
**Anti-** ***C. pecorum*** ** IgM**	**Low**	128	3.30^A^	3.27–3.33	2.92^A^	2.84–2.99	107.43^A^	95.20–119.66
	**High**	127	3.18	3.13–3.23	3.41	3.31–3.51	85.45	75.09–95.81
	**Low**	85	3.31^A^	3.27–3.35	2.80^A^^,^ B	2.72–2.89	116.06^A^^,^ b	101.18–130.94
	**Intermediate**	85	3.29^A^	3.24–3.34	3.15^A^	3.05–3.26	95.85	82.03–109.67
	**High**	85	3.13	3.07–3.19	3.53	3.42–3.63	77.54	65.03–90.04

# *C. pecorum* load: low <1.79, high ≥1.79; low <1.02, intermediate <2.62, high ≥2.62; anti-*C. pecorum* IgM: low <2173, high ≥2173; low <1270, intermediate <3636, high ≥3636.

Significant differences are indicated by.

a from “High” at *P*<0.05;

A from “High” at *P*<0.01;

b from “Intermediate” at *P*<0.05;

B from “Intermediate” at *P*<0.01.

To further identify possible physiological mechanisms that mediate the effect of chlamydial infection on growth rates, we analyzed a set of hematological parameters that could potentially also serve as surrogate markers for such mechanisms. While complete and differential blood counts and plasma iron did not show consistent differences, plasma albumin and globulin showed highly significant and consistent differences in dependence of chlamydial infection intensity (Table 2). Plasma albumin followed the pattern of weight gains and was highly significantly reduced at high chlamydial infection intensity. In contrast, plasma globulin showed the reverse pattern and followed the changes in conjunctival inflammation, with high values highly significantly associated with high chlamydial infection intensity.

These data support the notion that clinically inapparent chlamydial infection elicits an inflammatory response that is detrimental to growth in calves, consistent with the growth stunting effects of bacterial infections or LPS of gram-negative bacteria that is mediated by circulating inflammatory cytokines such as IL-1, IL-6, and TNF-α [41], [42]. Another possibility, however, would be that confounding factors that are collinear with the parameters of chlamydial infection simulate the observed effects. While controlling for breed, time of enrollment, or cohort size does not change the patterns of result, controlling for age or body weight of the calves reduces many effects substantially and eliminates some. In fact, chlamydial loads, anti-chlamydial IgM levels, and conjunctival inflammation increased with age, and concomitantly with body weight, of the calves (Fig. 1B, C, D). Simultaneously the body weight gains of the calves decreased with age (Fig. 1A), at a time when their nutrition was converted to roughage, and the calves had more frequent cohort contacts on the free-range pasture.

To circumvent the problem of collinearity, we used two approaches: 1) analyze week 1–5 data as control dataset, and 2) analyze a physiological marker of growth. Use of the week 1–5 dataset highly significantly confirmed that calves with high anti-*C. pecorum* IgM had lower weight gains than those with low IgM (12.40 vs. 17.38%, *P* = 0.003), despite the confounding influence of maternal antibodies and overall lower chlamydial infection intensity. Interestingly, in the week 1–5 dataset, age was highly significantly negatively correlated with anti-*C. pecorum* IgM (*P*<10^−4^), but consequently, in contrast to the week 7–15 dataset, positively correlated with weight gain. Therefore, body weight gains were consistently higher in calves with low anti-*C. pecorum* IgM, but did not consistently change with age of the calves.

To confirm this observation, we analyzed as direct marker of growth the plasma levels of insulin-like growth factor-1 (IGF-1), the systemic mediator of somatic growth [43]. Again, IGF-1 completely followed the pattern of body weight gains and plasma albumin, and the reverse patterns of chlamydial infection intensity, conjunctival inflammation, and plasma globulin. These data confirm that chlamydial infection intensity itself associates with reduction in actual somatic growth, expressed as decreased plasma IGF-1, rather than confounding variables simulate this outcome while the propensity for body weight gains remains constant.

### Multifactorial modeling of *C. pecorum* infection and host response

**Figure 2:**
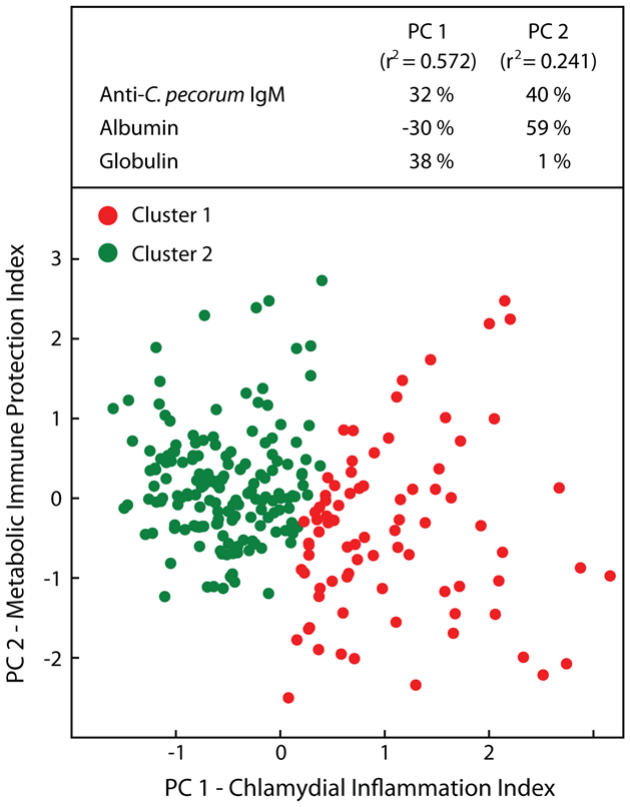
Multifactorial modeling of *C. pecorum* infection and host response by principal component and cluster analyses. Principal component analysis of the 255 observations of 51 calves from week 7 to 15 used the three parameters that best accounted for data variance: plasma levels of anti-*C. pecorum* IgM antibody, albumin, and globulin. More than 81% of variance observed among animals and between sampling points were explained only by two principal components that were termed to reflect the biological significance of the combination of the original variables as shown with their partial r^2^ in the upper panel. Cluster analysis of PCs for each data point separated all data into two clusters based on Euclidean distances between data points (cluster 1, n = 84; cluster 2, n = 171).

To analyze the complex interactions of *C. pecorum* infection and host response to it, we used a hypothesis-free modeling approach. **P**rincipal component analysis combined markers for chlamydial infection and host response to maximally explain the variance in the observations, subsequent cluster analysis grouped similar observations, and T-tests of clusters revealed the biological significance. Anti-*C. pecorum* IgM, albumin, and globulin combined into two principal components (PC) resulted in the greatest explanatory power of all potential combinations of original variables (r^2^ = 0.813; Fig. 2). PC 1 was composed in approximately equal parts by these 3 variables, with albumin negatively contributing, and explained 57% of the variance (r^2^ = 0.572). We interpreted the strong contribution of anti-chlamydial IgM to PC 1, as well as the negative albumin and positive globulin contribution, as an indicator of the inflammation driven by chlamydial infection, and therefore termed PC 1 “Chlamydial Inflammation Index”. PC 2 was positively affected by albumin and anti-*C. pecorum* IgM, but not globulin, and explained 24% of the variance (r^2^ = 0.241). Our interpretation of the positive anti-chlamydial antibody contribution combined with the positive albumin contribution was that this reflected immune protection following chlamydial infection that improved liver function. Therefore we termed PC 2 “Metabolic Immune Protection Index”.

Cluster analysis of these two principal components separated cases with high chlamydial inflammation index and low metabolic immune protection index as cluster 1 from cases with reverse index characteristics as cluster 2 (Fig. 2). Cluster 1 calves had a highly significantly lower biweekly weight gain of 9.31% as compared to cluster 2 calves with 17.82% weight gain, a profound 48% reduction of the growth rate (Table 3; *P*<10^−4^). This reduced weight gain was highly significantly accompanied by decreased albumin, IGF-1, and metabolic immune protection index, and increased conjunctival inflammation, *C. pecorum* load, anti-*C. pecorum* IgM, globulin, and chlamydial inflammation index (*P*<10^−4^). Interestingly, using these principal components to separate cases, plasma iron was also found highly significantly decreased in cluster 1 calves (*P* = 0.002).

**Table 3:** Physiological and chlamydial infection parameters categorized by PCA cluster.

	Cluster 1 (N = 84)^a^	Cluster 2 (N = 171)
**Biweekly Body Weight Gain, %**	9.31±2.04	17.82±1.37
**Conjunctival Inflammation Score**	2.55±0.14	2.13±0.05
***C. pecorum*** **/swab, Log_10_**	2.83±0.36	1.53±0.19
**Anti-** ***C. pecorum*** ** IgM, rlu/sec**	7379.46±1864.30	2001.70±331.75
**Iron, µg/dL**	145.94±9.82	168.37±8.53
**Albumin, g/dL**	3.00±0.05	3.36±0.02
**Globulin, g/dL**	3.65±0.10	2.92±0.06
**IGF-1, ng/mL**	74.30±13.18	107.38±9.84
**Chlamydial Inflammation Index**	1.096±0.17	−0.538±0.09
**Metabolic Immune Protection Index**	−0.349±0.25	0.172±0.13

a Data are shown ±95% confidence interval. Differences between cluster 1 and 2 are significant at *P* = 0.0017 for plasma iron, and at *P*<10^−4^ for all other parameters.

**Figure 3:**
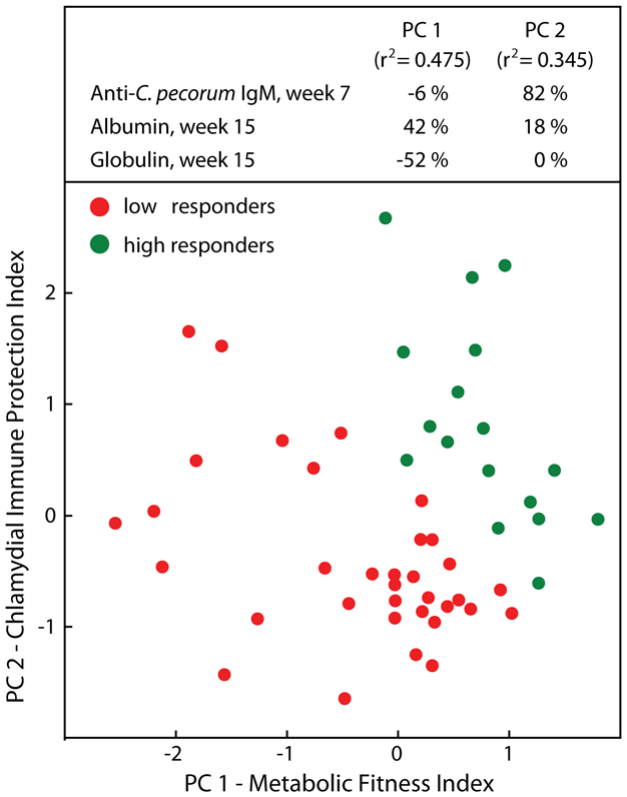
Modeling of metabolic health in dependence of earlier anti-*C. pecorum* immunity. Predictive modeling by principal component and cluster analysis of plasma albumin and globulin in week 15 of each animal combined with the corresponding anti-*C. pecorum* IgM 8 weeks earlier in week 7 generated two PCs with a combined r^2^ of 0.82. Two clusters of calves were termed low (n = 34) or high responders (n = 17) based on the values for each PC.

**Figure 4:**
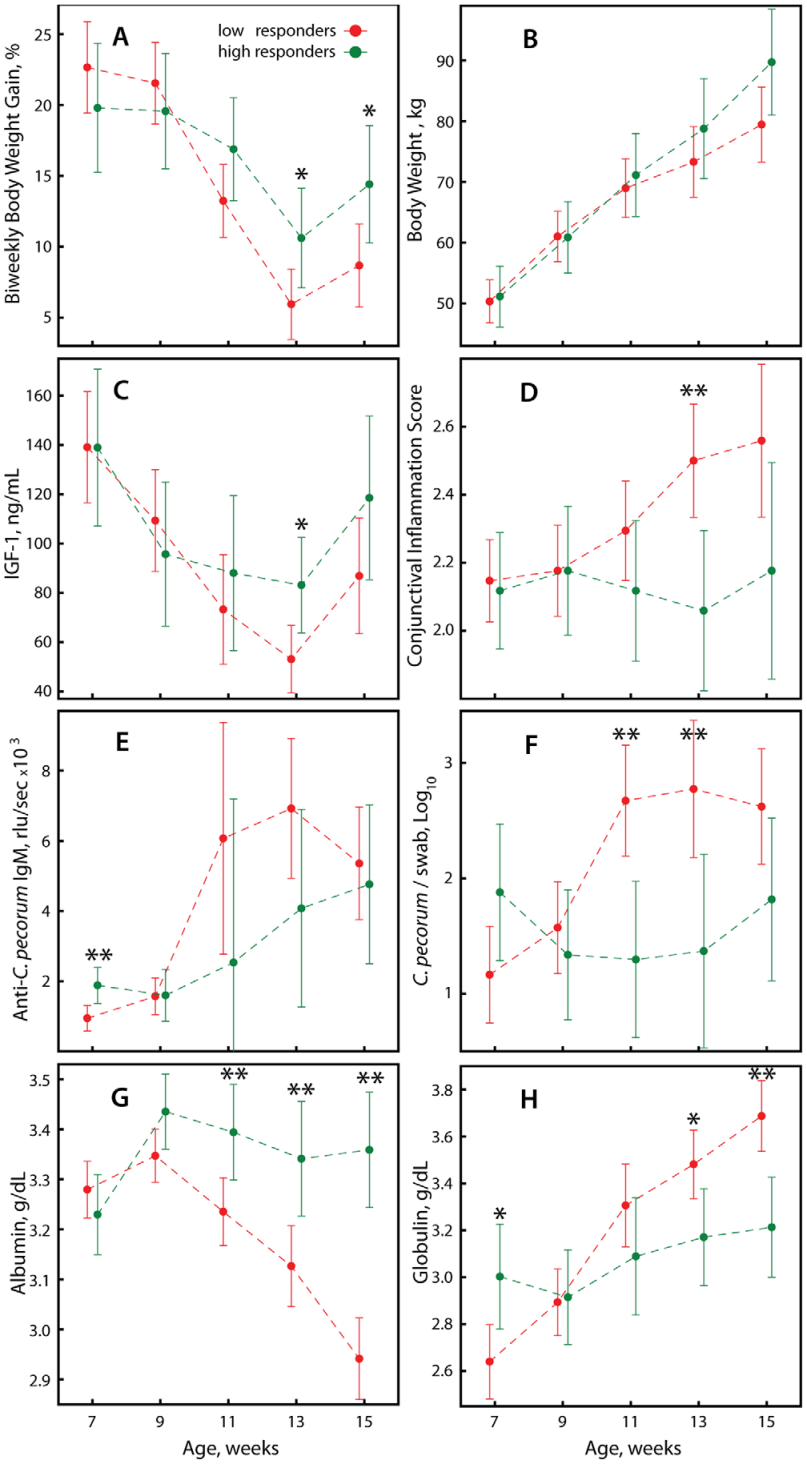
Growth, health, and chlamydial infection parameters based on modeling of metabolic health in dependence of earlier anti-*C. pecorum* immunity. The progression of low (n = 34) and high responder calves (n = 17) in Fig. 3 over the sampling period from week 7 to week 15 is shown. (A) Body weight gains. (B) Absolute body weight. (C) Plasma insulin-like growth factor-1. (D) Conjunctival inflammation. (E) Anti-*C. pecorum* IgM. (F) *C. pecorum* genomes per swab. (G) Plasma albumin. (H) Plasma globulin. For evaluation of statistical significances of differences between responders at sampling time points, data are shown ±95% confidence interval. *, *P*<0.05; **, *P*<0.01.

Anti-chlamydial IgM antibodies in our analyses thus far were used to indicate chlamydial infection intensity contemporaneously with health outcomes. In the next analysis, we asked if earlier chlamydial infection intensity could also act as a leading indicator of an anti-chlamydial immune response that protected against chlamydial infection and affected subsequent growth rates. To this end, we combined in principal component analysis anti-*C. pecorum* IgM in week 7 for each calf with plasma albumin and globulin data eight weeks later in week 15. Two PCs explained 82% of the variance (Fig. 3). Albumin contributed positively, and globulin negatively to PC 1, which was therefore termed “Metabolic Fitness Index” while PC2 was virtually exclusively positively driven by anti-*C. pecorum* IgM and was termed “Chlamydial Immune Protection Index”. Cluster analysis separated all calves into two clusters, which we termed low and high responders on the basis of high or low values in the PCs (Fig. 3). Calves of the high responder cluster started out in week 7 with body weight gains similar to low responders, but gradually developed increasing weight gains that were significantly higher in weeks 13 and 15 (Fig. 4A; *P*≤0.034). These higher weight gains translated from similar starting body weights to 10.3 kg higher body weight of 89.7 kg of high responders compared to 79.4 kg of low responders, albeit statistical significance was not reached (Fig. 4B; *P* = 0.059). IGF-1 followed a similar and was significantly higher in week 13 for the high responder cluster (Fig. 4C; *P* = 0.014). Clinically, the high responder cluster did not show conjunctival inflammation while the low responders developed increased inflammation over time, highly significantly so in week 13 (Fig. 4D; *P* = 0.003). These physiological differences were accompanied by corresponding trends in markers of chlamydial infection intensity (Fig. 4E, F) and plasma markers of inflammation and metabolic health (Fig. G, H). These outcomes were consistent with previous results (Table 1, 2, 3; Fig. 2) in that the healthier high responder calves over time showed reduced anti-*C. pecorum* IgM (Fig. 4E), *C. pecorum* loads (Fig. 4 F), plasma globulin (Fig. 4H), and increased plasma albumin (Fig. 4G). In summary, these data support the notion that *C. pecorum* infection leads to immune protection from disease or subclinical health consequences, but not to sterilizing immunity that immediately eliminates chlamydial infection.

## Discussion

The 100% prevalence of *C. pecorum* infection in individual calves over the study period, and the 74% prevalence at any given sampling time point, conform to other studies worldwide [12]. The detection of multiple strains strongly indicates endemic infection in which several distinct *C. pecorum* strains circulate in the herd. Interestingly, our data do not show an obvious reason such as herd immunity for the circulation of multiple *C. pecorum* strains. We observed largely primary infections of the calves, and the switch to different strains was not immune-driven, because some calves did show repeated infection peaks about 10 weeks after primary infection with either the same or a different strain. In addition, some calves had mixed infections or simultaneous infection with two or more strains at different mucosal sites.

The prevalence of the infection was particularly high after weaning at 7–8 weeks of age when the calves were moved from individual hutches in which they had no direct contact with each other to a common pasture with free movement of all animals. The high sampling intensity was instrumental in proving that every single animal of the cohort experienced an acute phase of the chlamydial mucosal infection over a 4–6 week period, preceded and followed by occasional detection of low-level chlamydial infection that presumably was immune-controlled. The detection of IgM antibodies against *C. pecorum* in every single calf over the study period is consistent with the ubiquitous presence of *C. pecorum* in the herd and the highly efficient colostral transfer of maternal IgM antibodies to the calf, the short 4-day half-life of these antibodies in the calf, and the calf's own emerging IgM response to the chlamydial infection [24], [38], [39], [40].

Despite the omnipresent *C. pecorum* infections, none of the study calves ever showed clinical signs of disease other than increased conjunctival redness that associated highly significantly with high chlamydial burdens or IgM antibody responses. In addition, none of the whole blood parameters included in the complete blood count as well as the plasma parameters analyzed deviated from accepted standard ranges [44], [45], [46].

Thus, in the present study, we encountered a scenario different from the observational studies that addressed asymptomatic bovine chlamydial herd infection by scoring for presence of absence of chlamydial infection. Every animal in this study experienced a course of a first natural chlamydial infection. Thus, we did not ask the question if presence or absence of chlamydial infection associated with changed health, but if changed intensity of chlamydial infection resulted in different health outcomes. The results clearly support the notion that an increased chlamydial infection causes a significant reduction of the growth rate of calves (Table 1), and that increased immunity after peak infection protects from growth suppression for at least 8 weeks (Fig. 4). These results are consistent with those of Reinhold et al. [29] who show that at enrollment in their study *Chlamydia*-infected calves had lower body weight than *Chlamydia*-non-infected calves. However, over the ensuing five study months the difference in body weight did not change in their study. Hematological parameters of *Chlamydia*-infected calves actually tended to relatively improve over the study period from lower values than in *Chlamydia*-non-infected calves. Thus in the study by Reinhold et al. [29], low-level chlamydial infection significantly associated with lower health at enrollment, but did not have a measurable negative effect on calf health over the study period. We ascribe this lack of a negative influence to a protective immune response to the chlamydial infection.

What is the mechanism of growth suppression by subclinical chlamydial infection? One explanation may be malabsorption of nutrients due to the local inflammatory response to intestinal mucosal chlamydial infection. While it is clear that *C. pecorum* resides in the intestinal tract of calves [9], [15], [24], clinical symptoms would have been evident in our study if *C. pecorum* had caused enteritis and/or malabsorption syndrome. In the absence of such symptoms, a more likely explanation is a systemic effect of the sum total of inflammatory mediators released in response to the inapparent *C. pecorum* infection of virtually all mucosal membranes. This would be similar to the exacerbation of insulin resistance in obese mice experimentally infected with *C. pneumoniae* that was mediated by circulating TNF-α released from the lung, the primary infection site [47].

To evaluate the possible role of chronic low-level systemic inflammation in the *C. pecorum*-associated growth depression, we analyzed albumin and globulin as global plasma markers of inflammation [48]. Albumin is exclusively produced by hepatocytes and inflammation reduces its synthesis, thus albumin is an inverse marker of the acute-phase response [49]. Globulin encompasses all remaining plasma proteins that include hepatocyte-produced proteins such as haptoglobin, but also immunoglobulins, and is a direct marker of the acute-phase response due to its increase during inflammation [49]. Because of their extended plasma half-life, these proteins are better markers for chronic inflammatory conditions than typical acute-phase proteins with short half-lives such as C-reactive protein [48], [50]. Both albumin and globulin highly significantly track *C. pecorum* load and anti-*C. pecorum* IgM levels in the anticipated patterns (Tables 2, 3; Fig. 4G, H), strongly suggesting that in fact it is the systemic inflammatory response, and in particular its detrimental effect on the liver, that mediate the growth depression at high, but clinically inapparent *C. pecorum* infection.

To further confirm the central role of the liver in chronic inflammation-mediated growth depression, and to address the collinearity of age, chlamydial infection and weight gains, we analyzed plasma insulin-like growth factor-1. IGF-1 is the actual mediator of somatic growth [43]. It is largely produced by hepatocytes, and inflammatory stimuli such as TNF-α, IL-1, or IL-6 reduce its synthesis and plasma levels [42]. Low plasma IGF-1 invariably results in reduced growth by uncoupling the somatotropic axis via induction of cellular resistance to growth hormone [41], [51]. Again, plasma IGF-1 was highly significantly decreased at high chlamydial infection and closely tracked growth rates, confirming that *C. pecorum* infection reduced growth rates of calves via an infection intensity-dependent liver response to *Chlamydia*-induced systemic inflammation (Tables 2, 3; Fig. 4C).

As any biological outcome, growth depression by chlamydial infection is not only influenced by environment and pathogen load, but also by the host genetics-driven response to the inflammatory insult. To account for both chlamydial and host factors, we analyzed the data by hypothesis-free principal component analysis considering both inputs, and separated the data into clusters of related cases (Fig. 2, Table 3). The weighted combination of input variables into principal components provided actual biological significance to the interaction of these variables by explaining that anti-chlamydial IgM immunity is both a marker for inflammation driven by chlamydiae (Chlamydial Inflammation Index, Fig. 2) as well as for the protective function of the immune response to chlamydiae (Metabolic Immune Protection Index, Fig. 2). Comparison of the clusters unambiguously showed that high chlamydial infection intensity highly significantly co-segregated with high plasma globulin and low albumin, IGF-1, and body weight gain. In fact, the difference of 48% in body weight gain was the highest of all contrast analyses in this study, and confirmed that consideration of both chlamydial infection and host response optimally models the outcome.

In a second PCA, grouping of individual animals into high and low responders based on the immune response in week 7 combined with the metabolic response in week 15 allowed us evaluate the potential for protection by the immune response to an earlier chlamydial infection. In this analysis, high immunity in week 7 highly significantly co-segregated with high metabolic fitness (high albumin, low globulin) in week 15 confirming that chlamydial infection mediates later immunity that protects from disease (reduced growth) but is not able to completely eliminate subsequent chlamydial infections (Fig. 3, 4). This immune protection resulted in week 15 in 66% increased growth rate (14.4 vs. 8.7% bi-weekly weight gain, *P* = 0.027) and 13% increased body weight (89.7 vs 79.4 kg, *P* = 0.059) in protected high-responder calves versus unprotected low-responder calves (Fig. 4A, B).

In summary, this investigation further establishes the negative health effects of endemic subclinical *C. pecorum* infections in cattle. These infections impact liver health and decrease plasma IGF-1 in calves resulting in reduction of somatic growth by up to 48%, while causing no signs of clinical disease other than subtle reddening of the conjunctiva. The worldwide detection of the omnipresence of these chlamydial infections may be still obscured by misleading serological assays [22], [27], [52] and the laborious and stochastic nature of PCR detection of low chlamydial burdens [14], [23]. However, highly sensitive chlamydial genus- and species-specific peptide ELISAs based on immunodominant proteins identified from all chlamydial genomes may soon be available and correct this shortcoming.

The findings reported here strongly suggest that anti-*C. pecorum* vaccination of calves within the first 4 weeks of life may be an effective way to increase growth similar to anti-chlamydial vaccination of cows that increases udder health [30]. In the context of the chlamydial depression of growth rates it is also of interest to contemplate a few facts: 1) antibiotics used as feed additives at sub-therapeutic concentrations consistently promote growth, and explanations for this effect center around poorly understood perturbations of the intestinal microbial flora [53], [54], [55]; 2) antibiotic feed additives are extensively used in animal agriculture and are still effective as growth promoters [56], [57], while many common bacteria have developed resistance against these antibiotics [57]; 3) chlamydiae can be found in any livestock operation; and 4) due to their sequestered intracellular habitat, chlamydiae are very inefficient at acquisition of antibiotic resistance, and the only antibiotic-resistant strains of chlamydiae with resistance acquired by horizontal gene transfer are strains of *C. suis* isolated from swine [58]. Could it be that in fact it is largely a suppression of chlamydial infections that is responsible for the growth promoting effect of antibiotics? In a scenario of growth depression by chlamydial infection, antibiotic growth promoters would be more effective in large herds and at high population density, poor hygiene and nutrition, and open herd management practices, essentially all situations that match actual observations on the effectiveness of antibiotic growth promoters [57]. Future vaccination against chlamydiae will test this hypothesis, and if successful, anti-chlamydial vaccines may partially or completely replace antibiotic feed additives as growth promoters.

## Materials and Methods

### Calves and calf husbandry

The study was performed at the EV Smith Dairy Unit of the Alabama Agricultural Experiment Station in Shorter, AL, USA. Dams were maintained in free-stall housing with mattresses, fed a 17% protein total mixed ration on corn silage base, and spent 6 hours per day on a grass lot. They were vaccinated once annually with a multivalent vaccine against bovine viral and bacterial diseases (Vira Shield 6+VL5 HB, Novartis Animal Health) and dewormed with doramectin (Dectomax, Pfizer Animal Health). Immediately after birth, calves were kept with the dam in a calving pen and fed colostrum of their dam or a colostrum pool. The next day, calves were separated from dams and housed in individual pens until they were weaned at 7 to 8 weeks of age. They received per day 4 kg bulk milk and water and 24% protein custom-mixed grain starter feed ad libitum containing 0.01% of the coccidiostatic lasalocid. Only healthy female calves born to dams that were free of any clinically apparent disease were enrolled in the study because male calves were removed from the herd at 2 weeks of age. After weaning, calves were raised in a common pasture with freely accessible hutches together with herd replacement heifers. In addition to seasonal grazing, they were provided hay ad libitum and 3 kg per day of starter calf compound feed containing 20% crude protein and 0.005% lasalocid. Throughout the study period, calves did not receive any antibiotics. Calf-dam herd health monitoring for bovine pathogens and herd health maintenance procedures were provided by the Auburn University Large Animal Clinic. All animal procedures were approved in protocol # 2010-1714 by the Auburn University Institutional Animal Care and Use Committee.

### Experimental design

The investigation was designed as prospective cohort study that examined the effect of natural infection with *Chlamydia* spp. on neonatal health and growth rates. In total, 26 Jersey and 25 Holstein female calves were continuously enrolled over a 25-week period and sampled for 39 weeks. Individual animals were sampled in 2-week intervals starting as early as on day 0 immediately after birth until 15 weeks of age. At each sampling time point, body weight was recorded and calves were scored for clinical parameters including alertness, muzzle dryness, conjunctival and vaginal mucosal color prior to swab sampling, lacrimal secretion, gait, and any signs of lameness. Mucosal color was recorded as inflammation score ranging from 1–4 (1 = white, anemic; 2 = normal pink; 3 = mild to moderate redness; 4 = pronounced redness). In addition, EDTA-blood, heparin plasma, and conjunctival and vaginal cytobrush specimens were collected from each calf.

### Hematological analyses

Blood was collected by venipuncture of the jugular vein in EDTA- and heparin-treated blood collection tubes (BD Vacutainer, Becton Dickinson and Company). Plasma was obtained from heparin blood by centrifugation at 1300×g for 15 min.

Complete blood counts were performed using an Advia 120 automated hematology analyzer following manufacturer's instructions (Siemens Medical Solutions). Complete blood counts and differential blood counts were recorded as absolute values and percentages, as well as morphologies of white blood cells (WBC), red blood cells (RBC) and platelets including left shift, atypical lymphocytes, blast cells, immature granulocytes, nucleated RBC, RBC fragments, RBC ghosts, platelet clumps, and large platelets.

### Plasma analyses

Plasma colorimetric chemistry analyses (iron, albumin, total protein) were performed by use of an automated cobas c 311 systems analyzer following manufacturer's instruction (Roche/Hitachi). Globulin was calculated as total plasma protein minus albumin. Plasma concentrations of IGF-1 were measured using a validated double antibody precipitation method as originally described [59]. Modifications to the assay as presently performed included the use of both human (100% homology to bovine) IGF-1 and rabbit antihuman IGF-1 serum (GroPep) as used for construction of the standard curve and as used as the primary antibody, respectively. Radioactive tracer for the assay was human ^125^I-IGF-1 (Perkin Elmer).

### Anti-*C. pecorum* immunoglobulin M (IgM) ELISA

*C. pecorum* type strain E58 (ATCC VR-628) [4] was propagated and elementary bodies (EB) purified as described [60], and EB lysates for use as antigen were prepared as described earlier [24]. Lysate antigen equivalent to 0.1 µg protein/well in bicarbonate buffer (15 mM Na_2_CO_3_, 35 mM NaHCO_3_, pH 9.6) was coated onto white, high protein binding flat-bottom microtiter plates. Plates were incubated overnight at 4°C, the coating solution aspirated, and wells washed 5 times with wash buffer (0.1 M Tris-HCl, pH 7.5, 0.5 M NaCl, 0.1% (v/v) Tween-20). Wells were then blocked with 200 µl of assay diluent (0.1 M Tris-HCl, pH 7.5, 0.5 M NaCl, 0.1% Tween-20, 10% (v/v) normal rabbit serum) for 1 hour at room temperature followed by 30 min incubation of 100 µl calf plasma diluted 1∶25 in assay diluent. After 5× washing, bound bovine IgM was detected with horseradish peroxidase-conjugated polyclonal sheep antibody against bovine IgM (Bethyl Laboratories), 1∶300 diluted in assay diluent, followed after 5× washing by incubation with chemiluminescent substrate (Roche Applied Science). The assay was read in a Tecan Spectrafluor Plus reader and results reported as relative light units per second (rlu/sec). The background signal for the negative control serum from a gnotobiotic calf (<500 rlu/sec) was subtracted from the data, and results were normalized between assay plates by a factor adjusting for differences between the signal of the positive control serum [3], [24].

### Quantitative detection of chlamydiae, *ompA* typing, and DNA sequencing

Conjunctival cytobrush swab samples were collected with a single swab from both eyes by extroverting the lower eyelid with thumb and index finger to form a pocket in which the cytobrush (Puritan, Hardwood Products) was rotated 5 times. The cytobrush handle was clipped, and the swab was immediately transferred into 400 µl RNA/DNA Stabilization Reagent for Blood/Bone Marrow (Roche Applied Science) in microcentrifuge tubes. Swab samples were centrifuged at 250×g for 1 min and stored at −80°C without the brush. Vaginal swab cytobrush samples were collected similarly after cleaning and alcohol disinfection of the perivaginal area.

Prior to nucleic acid extraction, all samples were homogenized (Precellys 24 tissue homogenizer/grinder; Bertin). After total nucleic acid extraction by glass fiber binding and elution in 2×20 µl Tris-EDTA buffer [61], chlamydial DNA was quantitatively detected and differentiated by modified real-time fluorescence resonance energy transfer (FRET) PCR targeting the *Chlamydia* spp. 23S rRNA gene [24], [61]. Primer CHL23SUP was replaced by primers CPEC23SUP (GGGGTTGTAGGGTCGATAACGTGAGATC) and CTR23SUP (GGGGTTGTAGGRTTGRGGAWAAAGGATC) which were each used at 0.5 µM final concentration. New probes used were genus-specific CHL23SFLU (GRAYGAHACAGGGTGATAGTCCCGTA-6FAM; 0.1 µM), *C. abortus-*, *C. psittaci-*, *C. pecorum-*, *C. pneumoniae-*, *C. felis-*, and *C. caviae*-specific CP23LCR (LightCycler Red 640-ACGAAARAACAARAGACKCTAWTCGAT-Phosphate; 0.2 µM) and *C. trachomatis-*, *C. suis-*, and *C. muridarum*-specific CTR23CY5.5 (Cy5.5-ACGAAAGGAGAKMAAGACYGACCTCAAC-Phosphate; 0.2 µM). This probe composition allowed detection of the CP23LCRF FRET signal at 640 nm and of the CTRCY5.5 signal at 705 nm. All other parameters remained unchanged. Confirmatory typing of chlamydial species was performed by chlamydial *ompA* real-time FRET PCR as described [61]. DNA sequencing of filter-purified *ompA* amplification products was performed with both primers by a fluorescent Sanger method (Eurofins MWG Operon). The DNA sequence of the novel *ompA* genotype Smith3v8 identified in this study has been deposited to GenBank with the accession # JX272924.

### Statistical Analyses

All statistical analyses were performed with the Statistica 7.0 software package (StatSoft). Chlamydial load data were logarithmically transformed after addition of 1 to the linear data. Negative results were treated as 0 in the log-transformed data. The average chlamydial load per calf at any sampling time point was calculated as the mean logarithm of vaginal load plus conjunctival load multiplied by the ratio of vaginal: conjunctival mean logarithm. The difference in PCR positivity of conjunctival and vaginal samples was evaluated by Chi square test.

Cases were separated by median values or 33.3 and 66.6 percentiles into ordinal categories of chlamydial loads or anti-*C. pecorum* IgM, or into clusters by cluster analysis of principal components. Principal component analysis (PCA) based on the correlation matrix was used to reduce the dimensionality of the data set and to identify principal components as linear combinations of optimally-weighted underlying original variables. The principal components were then used to perform a disjoint cluster analysis to delineate natural clusters present in the cases based on least-squares estimation of Euclidean distances. Effects between groups were measured using Student's t-test for normally distributed data as determined by Shapiro-Wilk's W test. Comparison of means of ordinal data such as the conjunctival inflammation score was performed by the non-parametric Mann-Whitney test.
